# Ubiquitin-specific protease 25: a new regulator for cardiovascular and cerebrovascular diseases

**DOI:** 10.3389/fcell.2026.1802539

**Published:** 2026-07-23

**Authors:** Yingchun Shao, Shan Jiang, Li Zhai, Fusheng Sun

**Affiliations:** Qingdao Hospital, University of Health and Rehabilitation Sciences (Qingdao Municipal Hospital), Qingdao, China

**Keywords:** cardiovascular diseases, cerebrovascular diseases, deubiquitinating enzyme, ubiquitin-specific protease 25, USP25

## Abstract

Ubiquitin-specific protease 25 (USP25), a pivotal deubiquitinating enzyme, has attracted increasing attention in cardiovascular and cerebrovascular research due to its regulatory function in maintaining ubiquitination homeostasis. Considerable advances have been achieved in elucidating the molecular mechanisms through which USP25 participates in the pathological progression of diverse cardiovascular and cerebrovascular disorders. From the perspective of translational medicine, notable progress has also been observed in the development of USP25-targeted therapeutics, with several inhibitors having advanced to preclinical and early-phase clinical trials, thereby offering promising targets for precision treatment. Nevertheless, a systematic synthesis of fundamental biological properties, the trajectory of research development, and the intricate upstream–downstream regulatory networks of USP25, together with its mechanistic roles and therapeutic potential in these diseases, remains absent. In response, this review provides an in-depth summary of the essential biological features of USP25, elucidates the interplay between its upstream regulators and downstream substrates, and highlights its specific molecular mechanisms in cardiovascular and cerebrovascular disease pathogenesis. Additionally, the feasibility and strategic approaches of targeting USP25 for therapeutic purposes are analyzed, while the limitations and potential breakthroughs in the development of USP25 inhibitors are discussed. Finally, priority research directions are proposed to guide future exploration, with the ultimate aim of contributing theoretical foundations and novel perspectives for clinical management of cardiovascular and cerebrovascular diseases.

## 1 Introduction

Ubiquitination represents a post-translational protein modification pathway (Popovic et al., 2014; Lacoursiere et al., 2022; Agrata and Komander, 2025). The conjugation and removal of ubiquitin are catalyzed by the ubiquitinase complex (E1, E2, and E3) and deubiquitinating hydrolases (DUBs), respectively (Deng et al., 2020; Dikic and Schulman, 2023; Zhang et al., 2024b). Ubiquitinated proteins are subsequently directed to the proteasome, which is responsible for their degradation, thereby constituting the ubiquitin–proteasome system (Grice and Nathan, 2016; Hu et al., 2018; Li et al., 2022). Notably, DUBs are capable of reversing ubiquitination, thereby imparting reversibility to this modification. Within this group, the ubiquitin-specific protease (USP) family constitutes the largest subclass of DUBs, accounting for more than half of all known DUBs (Chen et al., 2021; Becirovic et al., 2024; Wang et al., 2025a). The USP family exerts multifaceted and indispensable functions, acting as a central regulatory element in fundamental biological processes.

The human genome encodes approximately 56 USPs, among which ubiquitin-specific protease 25 (USP25) constitutes a significant member of this family (Kim et al., 2015; Li and Ma, 2025). With respect to expression, USP25 exhibits high abundance in human skeletal muscle, cardiac muscle, testis, and brain. Functionally, USP25 has been implicated in cancers (Ye et al., 2025b), neurodegenerative disorders (Hu et al., 2024; Kim et al., 2024), gut (Li et al., 2025), cardiovascular diseases (Ye et al., 2023), and infectious diseases (Fu et al., 2025), indicating its potential as a therapeutic target across diverse pathological contexts. Mechanistically, USP25 selectively hydrolyzes K48- and K63-linked polyubiquitin chains, thereby modulating protein stability, subcellular localization, and activity (Zhu et al., 2021b; Santelices et al., 2025b). Through these molecular properties, USP25 is intimately connected to the onset and progression of a spectrum of diseases. Therefore, the development of USP25-centered therapeutic strategies is considered a highly promising avenue for the treatment of such conditions.

In 2024, PBSS-1113, a small-molecule USP25/28 inhibitor, entered a phase I clinical trial (CTR20220343) in hematologic malignancies, including acute myeloid leukemia (AML), to evaluate its safety, tolerability, pharmacokinetics, and preliminary antitumor activity. Early findings indicated that PBSS-1113 monotherapy exhibited antileukemic activity in patients with relapsed/refractory AML, with myelosuppression as the main treatment-related adverse event, which was reported to be manageable and reversible. In 2025, PBSS-1113 advanced into a phase II clinical trial (CTR20253847) to assess the safety and tolerability of PBSS-1113 plus azacitidine in patients with AML or myelodysplastic syndromes (MDS) and to determine the maximum tolerated dose (MTD) and recommended phase II dose (RP2D) of the combination regimen, supporting its further clinical development in myeloid malignancies. Moreover, AZ1 and CT1113 represent prototypical USP25/28 inhibitors, whose anti-inflammatory, anti-tumor, and Alzheimer’s disease (AD)-modifying effects have been substantiated in preclinical models. Nevertheless, investigations into USP25 within cardiovascular and cerebrovascular disorders remain at a pivotal transitional stage from fundamental research to clinical application, and no study has yet systematically synthesized or evaluated its functions in this context. Therefore, this review endeavors to provide a comprehensive account of the molecular structure, developmental milestones, regulatory networks, and specific biological functions of USP25 in cardiovascular and cerebrovascular diseases, with the purpose of constructing a translational medicine framework that spans target validation to drug development, thereby establishing theoretical and experimental foundations for innovative therapeutic strategies targeting these diseases.

## 2 Biological characteristics

**FIGURE 1:**
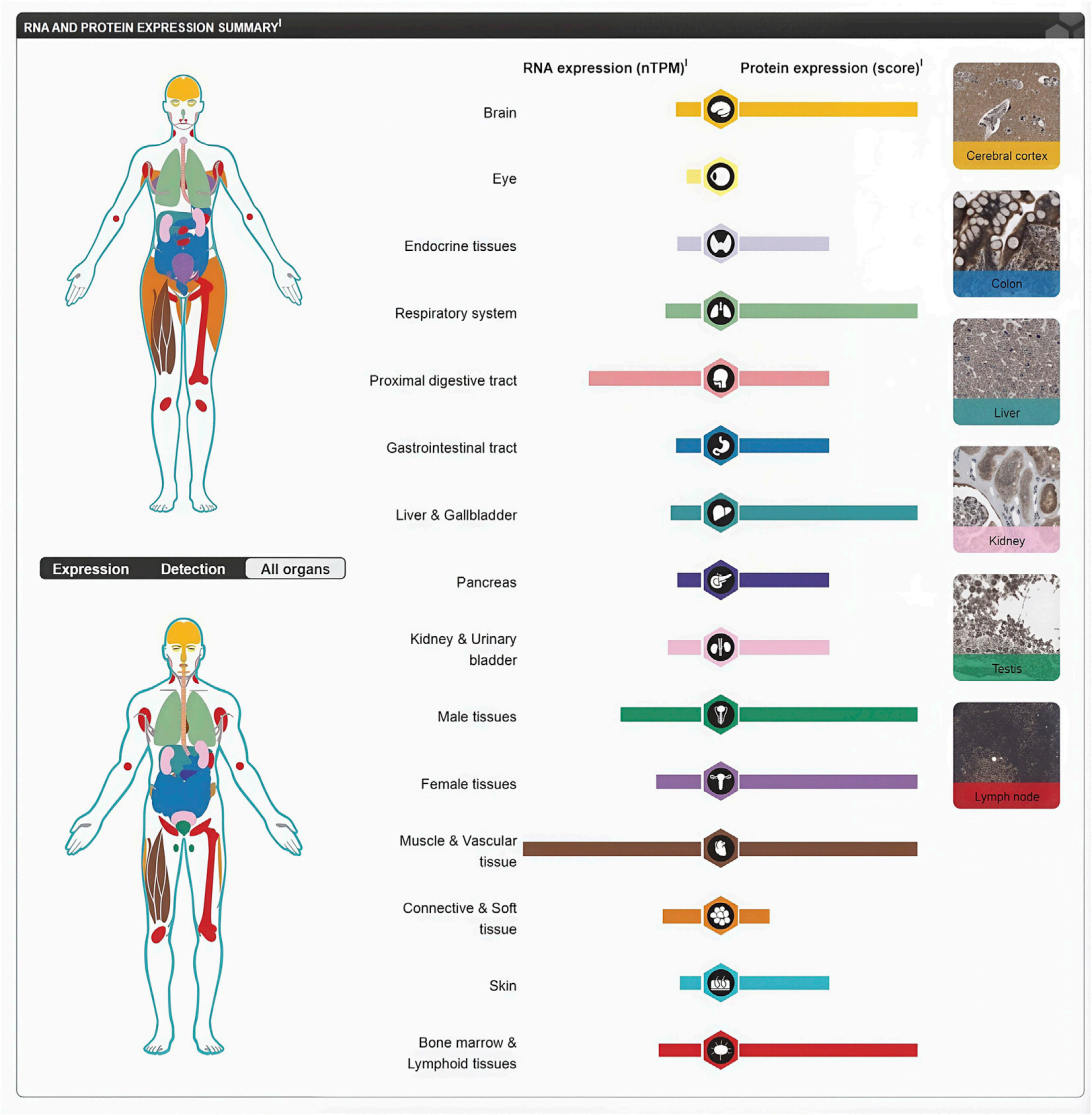
Expression of USP25 RNA and protein in different tissues and organs. The protein expression of USP25 is higher in the cerebellum, lung, gallbladder, testis, heart muscle, skeletal muscle, lymphnode, and spleen. The mRNA expression of USP25 is higher in the tongue, testis, and skeletal muscle. (https://www.proteinatlas.org/ENSG00000155313-USP25/tissue).

**FIGURE 2:**
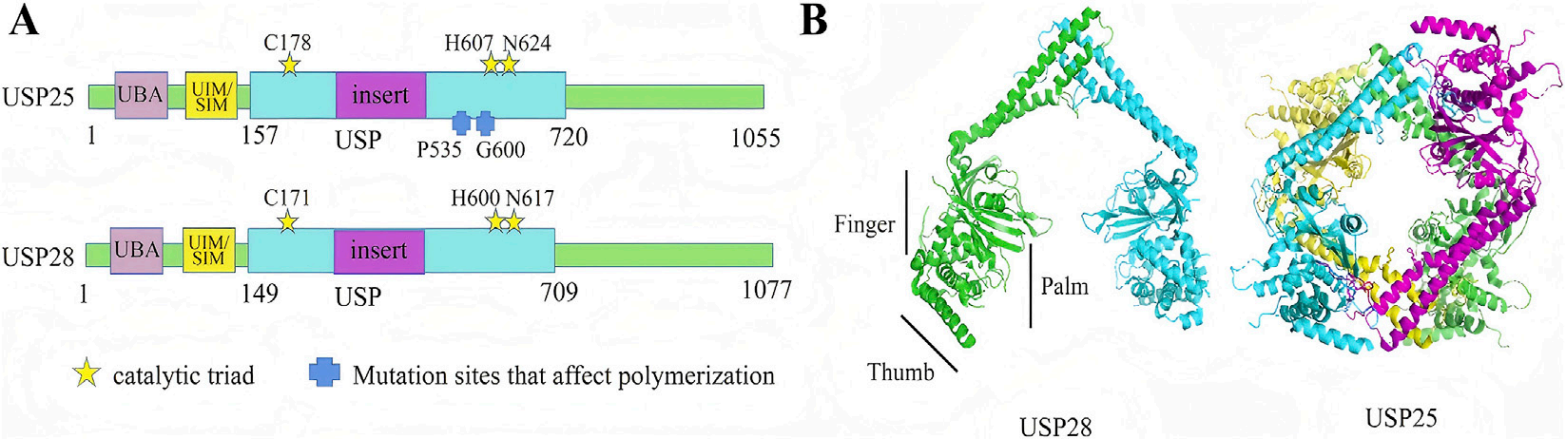
Schematic comparison of the structures of USP25 and USP28. **(A)** Schematic representation of the domain organization of USP25 and USP28. **(B)** Comparison of the three-dimensional conformations of the catalytic domains of USP25 and USP28 (Zhou et al., 2023). Copyright 2023, Elsevier. UBA, ubiquitin-associated domain; UIM, ubiquitin-interacting motif; SIM, SUMO-interacting motif; AIM, Auto-inhibited motif.

The USP25 gene is located on human chromosome 21 (21q11.2) and comprises 25 exons (Valero et al., 1999). Through alternative splicing, it generates three isoforms (USP25a, USP25b, and USP25m) (Habtemichael et al., 2018). The expression level of USP25 protein is relatively high in the cerebellum, lung, gallbladder, testis, myocardium, skeletal muscle, lymph nodes, and spleen (Figure 1). Compared with other DUBs, USP25 exhibits distinct enzymatic properties and substrate specificity, enabling the selective removal of ubiquitin chains conjugated to substrate proteins, particularly K63- and K48-linked ubiquitin chains (Shi et al., 2014; Wen et al., 2019). Notably, its enzymatic activity is further modulated by phosphorylation, SUMOylation, and ubiquitination (Meulmeester et al., 2008; Kim et al., 2015; Qian et al., 2018). Additionally, USP28 functions as the paralog of USP25. These two genes display high similarity not only at the genomic but also at the protein sequence level (Gersch et al., 2019; Patzke et al., 2024). As shown in Figure 2, USP25 and USP28 share a highly similar domain architecture, including an N-terminal ubiquitin-binding region (UBR), a ubiquitin-associated domain (UBA), a ubiquitin-interacting motif (UIM), a SUMO-interacting motif (SIM), the catalytic USP domain, and a C-terminal crimped domain. Their catalytic cores are also structurally well conserved. Notably, USP25 contains an auto-inhibited motif (AIM)-related autoinhibitory element that is absent in USP28 and can adopt an autoinhibited conformation through specific oligomerization. Consistent with this structural similarity, most currently reported USP25 inhibitors also target USP28 (Wrigley et al., 2017; Xu et al., 2023; Zhou et al., 2023), indicating that the high homology of their catalytic domains poses a major challenge for inhibitor selectivity. Therefore, the structural and functional similarities and differences between USP25 and USP28 are summarized in Table 1. As shown in Table 1, although USP25 and USP28 are highly homologous in sequence and catalytic domain organization, they differ in oligomerization-dependent regulation, substrate spectrum, and disease-associated functions. USP25 contains an AIM motif absent in USP28 and can form an autoinhibited tetramer, suggesting that its activity is more dependent on conformational regulation. Functionally, USP25 is more closely involved in inflammation, immunity, metabolism, and cardiovascular or cerebrovascular pathological remodeling, whereas USP28 mainly promotes tumor development by stabilizing oncogenic proteins such as c-MYC, c-JUN, NOTCH1, HIF-1α, and lysine-specific demethylase 1 (LSD1). Moreover, the role of USP28 in non-neoplastic diseases has also been preliminarily reported. For example, it may exert regulatory effects in pathological processes such as cardiac hypertrophy and diabetic cardiomyopathy (Han et al., 2024; Xie et al., 2024). These differences suggest that, although USP25 and USP28 belong to a family of highly homologous deubiquitinating enzymes, their structural basis and functional outputs do not completely overlap, thereby providing an important basis for selective drug design. Therefore, in the development of USP25-targeted drugs should consider not only the catalytic domain, but also USP25-specific features, including the AIM motif, oligomerization state, and substrate-interaction interfaces, to improve selectivity over USP28.

**TABLE 1:** Comparison between USP25 and USP28.

Characteristics	USP25	USP28	References
Chromosome localization	21q11.2	11q23	Valero et al. (2001)
Exon count	25	25	Valero et al. (2001)
Molecular weight	118 kDa	122.4 kDa	Valero et al. (2001)
Quantity of amino acids	1,055	1,077	Ruiz et al. (2021)
Sequence consistency	The overall sequence consistency is 51%, and the catalytic domain consistency is 57%	The overall sequence consistency is 51%, and the catalytic domain consistency is 57%	Gersch et al. (2019)
Domain composition	The N-terminal contains domains such as UBA, UIM, and SIM, the USP catalytic domain, and the UCID insertion domain (AIM,^187^RRLVLS^192^), while the C-terminal is a curly domain	Similar to USP25, the N-terminal contains the UBA, UIM, and SIM domains, the USP catalytic domain, the UCID insertion domain (without AIM motif), and the C-terminal domain	Zhou et al. (2023)
Unique domain	Unique AIM motif	There are conformational differences in the α5 helix (residues 255-278) of the thumb subdomain of USP28 that are not present in USP25	Wrigley et al. (2017)
Key differences in catalytic domains	Blocking loop 1 (BL1) contains Arg385 and forms a salt bridge with BL2. The switching ring (SL) is short and highly rigid	BL1 contains Gln378 and has no salt bridge. SL is relatively long and highly flexible	Sauer et al. (2019)
Key sites for active regulation	AIM motif (Pro521/Phe522), BL1 (Arg385)	There are no key regulatory sites and the activity is stable	Sauer et al. (2019)
Oligomerization state	Dynamic equilibrium of dimer (active) - tetramer (self-inhibitory)	Stable dimer	Sauer et al. (2019)
Organizational distribution preference	Widely expressed, and highly expressed in cardiomyocytes and adipocytes	Highly expressed in tumor tissues such as colorectal and lung (tissues)	Xu et al. (2023)
Evolutionary conservation	The AIM motif is highly conserved in vertebrates	Lacks the AIM motif, while the dimerization interface is conserved	Gersch et al. (2019)
Cleavage	No relevant cleavage regulation	Cleavage by Caspase-8 leads to the loss of UBA and UIM domain fragments, resulting in protein inactivation	Zhou et al. (2023)
Key binding residues of inhibitors	E373, Q322, H268, and F299	E366, Q315, H261, and F292	Patzke et al. (2024)
IC_50_ (Ub-Rh110)	AZ1: 1.08 μM; VSM: 2.92 μM; FT206: 1.01 μM	AZ1: 1.76 μM; VSM: 3.51 μM; FT206: 0.15 μM	Patzke et al. (2024)
Inhibition mode	Non-competitive and reversible binding; efficient inhibition requires the disruption of tetramerization	Non-competitive and reversible binding, which directly blocks ubiquitin binding	Xu et al. (2023)
Intrinsic action of inhibitors	Competitive activation (relief of autoinhibition) + inhibitory activity	Pure inhibition (blocking substrate binding)	Patzke et al. (2024)
Core substrates	Mainly involved in inflammation, fibrosis, and cardiovascular remodeling	More closely associated with oncoprotein stabilization and tumor-promoting factors	Jiang et al., 2024; Zhang (2024), Wang et al. (2025c)
Associated diseases	Associated with Alzheimer’s disease, viral or bacterial infections, inflammatory disorders, metabolic dysregulation, and cardiovascular/cerebrovascular diseases	Closely associated with multiple malignancies, including colorectal cancer, lung cancer, squamous cell carcinoma, and breast cancer	Wang et al. (2018), Sadler et al. (2019), Wang et al. (2020)

UBA, ubiquitin-associated domain; UIM, ubiquitin-interacting motif; SIM, SUMO-interacting motif; AIM, Auto-inhibited motif.

## 3 Research timeline of USP25

**FIGURE 3:**
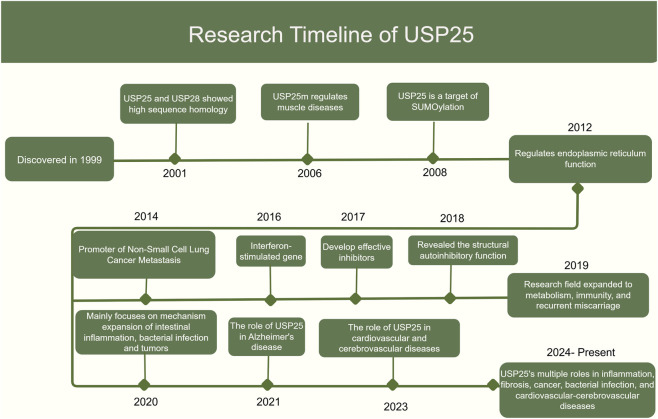
Research timeline of USP25. This figure depicts the timeline of discoveries related to the function of USP25 under physiological and pathological conditions, starting from 1999 until the present.

Research on USP25 commenced in 1999 with its initial identification and designation by Valero et al. (Valero et al., 1999). Sequence homology between USP25 and USP28 was reported in 2001 (Valero et al., 2001). In 2006, the muscle-specific isoform USP25m was discovered to interact with the sarcomeric proteins alpha-actin 1-encoding gene (ACTA1), Filamin C (FLNC), and muscle myosin-binding protein-C (MyBPC1), thereby implicating it in muscle disorders (Bosch-Comas et al., 2006). By 2008, USP25 was recognized as a target of SUMOylation (Meulmeester et al., 2008; Mohideen and Lima, 2008). In 2009, critical domains and substrate recognition mechanisms were delineated (Denuc et al., 2009). In 2012, USP25 was demonstrated to regulate endoplasmic reticulum function (Blount et al., 2012), and its contribution to inflammatory responses was further characterized in 2013 (Zhong et al., 2013a; Zhong et al., 2013b). A major advance occurred in 2014 when USP25 was implicated in promoting metastasis in non-small cell lung cancer (Li et al., 2014). In 2016, it was classified as an interferon-stimulated gene (Ren et al., 2016), thereby facilitating investigations into immune-associated pathways. From 2017, research shifted toward upstream regulatory mechanisms (Kawaguchi et al., 2017) and the development of inhibitors (Wrigley et al., 2017). In 2018, new insights were provided into inflammatory mechanisms (Long et al., 2018; Qian et al., 2018) and structural autoinhibition (Liu et al., 2018). Studies in 2019 expanded into functional comparisons with USP28 (Gersch et al., 2019; Sauer et al., 2019) and extended to metabolic diseases (Sadler et al., 2019), immunology (Wen et al., 2019), and recurrent miscarriage (Ding et al., 2019). In 2020, attention was directed toward intestinal inflammation (Wang et al., 2020), bacterial infection (Long et al., 2020), and cancer (Nino et al., 2020). The potential involvement of USP25 in AD was preliminarily investigated in 2021 (Zheng et al., 2021). In 2022, research further advanced into therapeutic strategies for AD (Zheng et al., 2022), inflammation (Chang et al., 2022; Liu et al., 2022; Lv et al., 2022), cancer (Dalland et al., 2022; Nelson et al., 2022), and asthma (Gan et al., 2022), while also identifying key targets in pancreatic cancer and providing additional clarification of structural autoinhibition. In 2023, functional studies of USP25 expanded to cardiovascular and cerebrovascular diseases (Li et al., 2023c; Ye et al., 2023). Since 2024, research has entered a stage of systematic integration, emphasizing its multifaceted roles in inflammation (Xu et al., 2024), fibrosis (Jiang et al., 2024), cancer (Zhu et al., 2024), bacterial infection (Fu et al., 2025), and cardiovascular diseases (Chen et al., 2025), thereby furnishing a comprehensive theoretical basis for understanding its pathophysiological roles and advancing targeted therapeutic strategies. The above main findings are summarized in Figure 3.

## 4 Upstream and downstream regulatory factors of USP25

**FIGURE 4:**
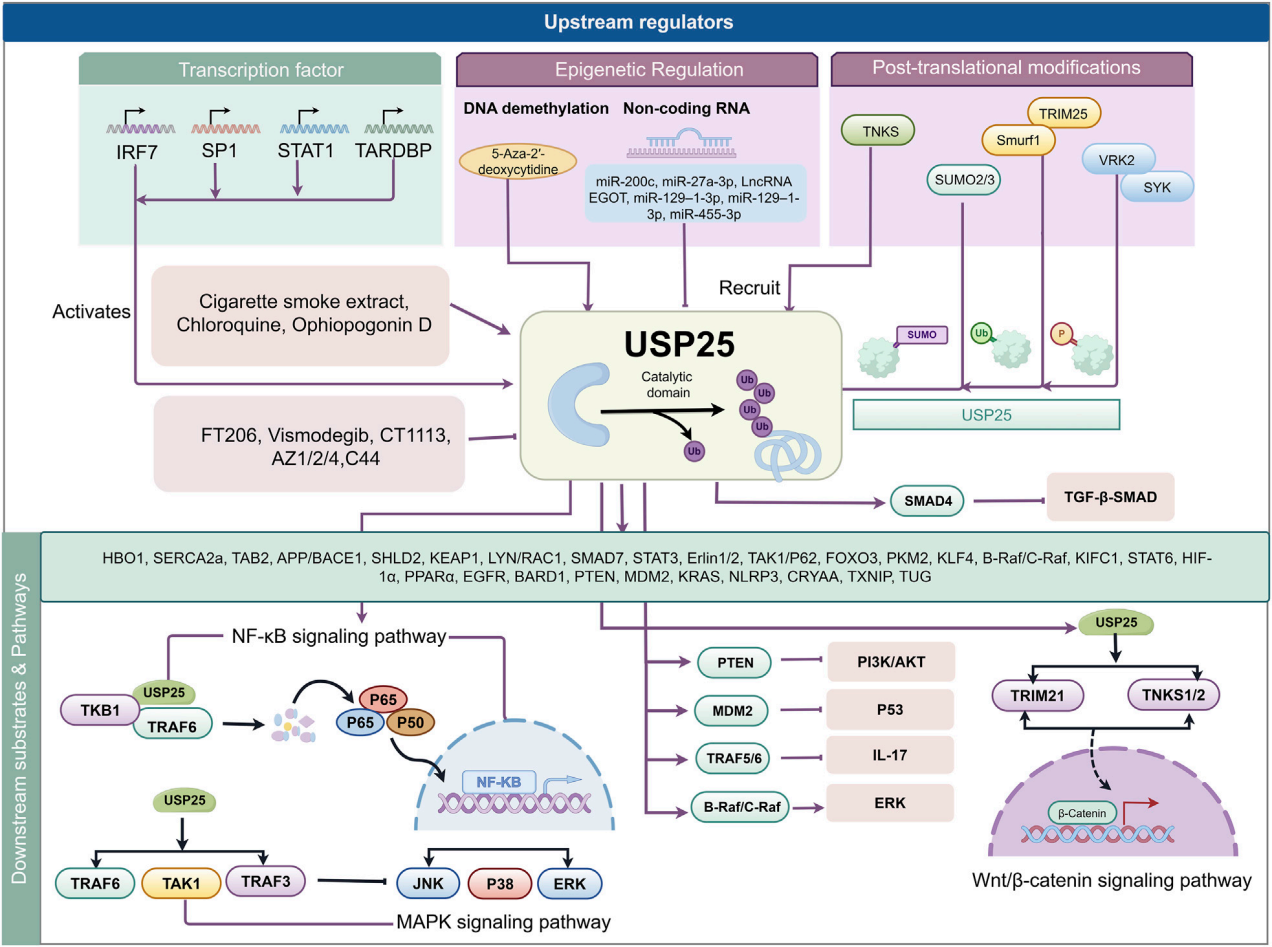
Integrated overview of the upstream regulators and downstream targets of USP25. The schematic illustrates the relationships among upstream regulatory factors, USP25, and its downstream substrates/signaling pathways. IRF7, Interferon regulatory factor 7; SP1, Specific protein 1; STAT1, Signal transducer and activator of transcription 1; TARDBP, TAR DNA-binding protein; TNKS, Tankyrases, SUMO2/3, Small ubiquitin-like modifier (SUMO) 2 and 3; Smurf1, Smad ubiquitylation regulatory factor 1; TRIM25, Tripartite motif-containing 25; VRK2, Vaccinia-related kinase 2; SYK, Spleen tyrosine kinase; HBO1, Histone acetyltransferase binding to the origin recognition complex 1; KEAP1, Kelch-like ECH-associated protein 1; Erlin1/2, ER lipid raft-associated 1 and 2; PKM2, Pyruvate kinase M2; KLF4, Kruppel-like factor 4; KIFC1, Kinesin family member C1; BARD1, BRCA1-associated RING domain 1; PTEN, Phosphatase and tensin homolog; MDM2, Murine double minute 2; CRYAA, AlphaA-crystallin; TXNIP, Thioredoxin interacting protein; TKB1, TANK binding kinase 1; TRAF6, TNF receptor-associated factor 6; TRIM21, Tripartite motif-containing protein 21; TNKS1/2, Tankyrase 1 and 2.

USP25 not only exerts a central regulatory role within the nucleus but also demonstrates substantial nucleocytoplasmic shuttling capacity. This dynamic subcellular localization enables extensive involvement in, and precise modulation of, the pathological processes underlying diverse diseases. Within this framework, factors regulating USP25 expression have exhibited considerable potential for clinical translation. Accordingly, Table 2 systematically summarizes the upstream regulatory factors of USP25, while Table 3 comprehensively integrates its downstream substrates and associated signaling networks. In addition, to more intuitively illustrate the hierarchical relationships and functional connections among the upstream regulators of USP25, USP25 itself, and its downstream targets, an integrated schematic diagram has been further provided (Figure 4), thereby establishing an overall framework of “upstream regulation–USP25–downstream effects.” This multidimensional framework, spanning from upstream regulatory mechanisms to downstream effector targets, is intended to provide a solid theoretical basis and data support for systematically evaluating the clinical translational potential of USP25.

**TABLE 2:** Upstream regulatory factors of USP25.

Regulatory type	Specific factors	Regulatory direction	Disease	References
Transcription factor	IRF7	Positive regulation	Virus infection	Ren et al. (2016)
	SP1	Positive regulation	Down syndrome/Alzheimer’s disease	Li et al. (2023b)
	STAT1	Positive regulation	Virus infection	Du et al. (2025)
	TARDBP	Positive regulation	Glioblastoma	Kaluzinska-Kolat et al. (2023)
Epigenetic regulation	5-Aza-2′-deoxycytidine	Positive regulation	L haplogroup cybrids	Atilano et al. (2022)
Protein	SUMO2/3	Sumo-modified	HeLa	Meulmeester et al. (2008)
	Smurf1	Ubiquitinated	Virus infection	Qian et al. (2018)
	TNKS	Recruit USP25	Insulin resistance	Sadler et al. (2019)
	TRIM25	Negative regulation	Colorectal cancer	Li et al. (2024)
	VRK2	Phosphorylated	Neurodegenerative diseases	Aslani et al. (2022)
	SYK	Phosphorylated	Hek293 cells	Cholay et al. (2010)
Non-coding RNA	miR-200c	Negative regulation	Non-small cell lung cancer	Li et al. (2014)
	miR-27a-3p	Negative regulation	Recurrent miscarriage	Ding et al. (2019)
	LncRNA EGOT	Negative regulation	Papillary thyroid carcinoma	Zhao et al. (2024)
	miR-129–1-3p	Negative regulation	Acute kidney injury	Huang et al. (2025)
	miR-200c	Negative regulation	Influenza A virus	Xu et al. (2022a)
	miR-455-3p	Negative regulation	Alzheimer’s disease	Kumar and Reddy (2018)
	miR-200b/200c/429	Negative regulation	Lung adenocarcinoma	Yin and Yu (2019)
Drug	Cigarette smoke extract	Negative regulation	Smoking-related lung diseases	Long et al. (2020)
	Chloroquine	Positive regulation	Sepsis	Ding et al. (2017)
	Ophiopogonin D	Positive regulation	Acute Lung Injury	Pu et al. (2025)
Inhibitor	FT206	Negative regulation	Pancreatic Ductal Adenocarcinoma	Nelson et al. (2022)
	Vismodegib	Negative regulation	Colorectal cancer	Wang et al. (2021)
	CT1113	Negative regulation	Liver injury/KRAS-driven cancer	Cai et al. (2023a), Ma et al. (2025)
	AZ1/2/4	Negative regulation	Alzheimer’s disease/Bacterial infection/Colorectal cancer/PH-positive leukemia/Non-alcoholic fatty liver disease	Shibata et al. (2020), Wang et al. (2020), Zheng et al. (2022), Liu et al. (2024), Santelices et al. (2025a)
	C44	Indirect competition	Prostate cancer	Cheng et al. (2019)

IRF7, Interferon regulatory factor 7; SP1, Specific protein 1; STAT1, Signal transducer and activator of transcription 1; TARDBP, TAR DNA-binding protein; TNKS, tankyrases, SUMO2/3, Small ubiquitin-like modifier (SUMO) 2 and 3; Smurf1, Smad ubiquitylation regulatory factor 1; TRIM25, Tripartite motif-containing 25; VRK2, Vaccinia-related kinase 2; SYK, spleen tyrosine kinase.

**TABLE 3:** Downstream substrates and signal regulatory networks of USP25.

Type	Downstream targets	Regulatory mechanism	Disease	USP25 functions	References
Protein	KIFC1	Direct combination	Cervical cancer	USP25 promotes the progression of cervical cancer	Ye et al. (2025b)
	SERCA2a	Direct combination	Myocardial hypertrophy	USP25 inhibited cardiac hypertrophy	Ye et al. (2023)
	B-Raf/C-Raf	Direct combination	Tuberculosis	USP25 enhances the anti-tuberculosis ability of macrophages	Fu et al. (2025)
	TUG	Direct combination	Diet-induced insulin resistance	USP25m enhances glucose uptake	Habtemichael et al. (2018)
	TRAF3	Direct combination	Endotoxin tolerance	USP25 inhibits inflammatory responses	Wen et al. (2019)
	HBO1	Direct combination	Bacterial infection	USP25 promotes LPS-induced inflammatory responses	Long et al. (2018)
	EGFR	Direct combination	EGFR -dependent tumor	The USP25-EGFR axis is positively correlated with the malignancy degree of tumors	Nino et al. (2020)
	APP/BACE1	Direct combination	Alzheimer’s Disease (AD)	USP25 aggravates the pathology of AD	Zheng et al. (2022)
	TBK-1	—	Acute pancreatitis (AP)	USP25 improves AP	Liu et al. (2022)
	KLF4	Direct combination	AP	USP25 promotes AP inflammation	Lv et al. (2022)
	TRAF3	Direct combination	Allergic rhinitis	USP25 inhibits the progression of allergic rhinitis	Chang et al. (2022)
	HIF-1α	Direct combination	Pancreatic ductal adenocarcinoma	USP25 promotes the progression of pancreatic ductal adenocarcinoma	Nelson et al. (2022)
	BARD1	Direct combination	Allergic asthma induced by dust mites	USP25 has a protective effect on dust mite-induced allergic asthma	Gan et al. (2022)
	TAB2	Direct combination	Ischemic stroke	USP25 reduces ischemic stroke injury	Li et al. (2023c)
	RORγt	—	Anti-glomerular basement membrane glomerulonephritis	USP25 inhibits excessive immune activation	Xu et al. (2024)
	LYN/RAC1	Direct combination	IgG4-RD	USP25 alleviates IGG4-related diseases	Jiang et al. (2024)
	FOXO3	Direct combination	Hypertension	USP25 improves vascular remodeling	Chen et al. (2025)
	SHLD2	Direct combination	Colorectal cancer (CRC)	USP25 promotes chemotherapy resistance in CRC	Li et al. (2024)
	KEAP1	Direct combination	Liver injury induced by acetaminophen	USP25 exacerbates drug-induced liver injury	Cai et al. (2023a)
	NRF2	Indirect effect	Liver injury induced by acetaminophen	USP25 exacerbates drug-induced liver injury	Cai et al. (2023a)
​	KRAS	Direct combination	Kras-driven cancer	USP25 promotes carcinogenic signaling of KRAS	Ma et al. (2025)
	BCR-ABL	—	Ph-positive leukemia	USP25 promotes the malignant proliferation of leukemia cells	Shibata et al. (2020)
	PPARα	Direct combination	Nonalcoholic fatty liver disease	USP25 inhibits the progression of nonalcoholic fatty liver disease	Liu et al. (2024)
	TRIM21	Direct combination	Hepatocellular Carcinoma (HCC)	USP25 promotes the progression of HCC	Liu et al. (2023b)
	TRAF6	Direct combination	Diabetic Kidney Disease (DKD)	USP25 improves DKD	Liu et al. (2023a)
	SMAD7	Direct combination	Type 2 diabetic nephropathy	USP25 alleviates renal pathological changes in type 2 diabetic mice	Wang et al. (2025b)
	STAT3	Direct combination	AP	USP25 exacerbates AP progression	Liu et al. (2021)
	Erlin1/2	Direct combination	Viral infection	USP25 restricts the replication and spread of RNA viruses	Teo et al. (2023)
	TAK1/P62	Direct combination	Obesity cardiomyopathy	USP25 improves obesity cardiomyopathy	Ye et al. (2024)
	PKM2	Direct combination	Acute kidney injury (AKI)	USP25 exacerbates AKI	Yang et al. (2023b)
	ATP5A1/ATP5B	Direct combination	Allogeneic organ transplantation	USP25 exacerbates transplant rejection	Li et al. (2023a)
	STAT6	Direct combination	Liver/lung fibrosis	USP25 promotes liver/lung fibrosis	Xu et al. (2025)
	PPARα	Direct combination	Metabolic dysfunction-Associated steatotic Liver disease (MASLD)	USP25 alleviates MASLD	Jin et al. (2025)
	PTEN	Direct combination	Polycystic ovary syndrome	USP25 promotes the progression of polycystic ovary syndrome	Gao et al. (2021)
	TNKS1/2	Direct combination	CRC	USP25 promotes the growth of tumor cells	Xu et al. (2017)
	MDM2	Direct combination	Diffuse large B-cell lymphoma	USP25 promotes the growth of tumor cells	Yang et al. (2023a)
	TRAF3/TRAF6	Direct combination	Viral infection	USP25 enhances the host’s antiviral immunity	Lin et al. (2015), Du et al. (2025)
	TRAF6	—	Acute lung injury	USP25 alleviates acute lung injury	Xu et al. (2022b)
	RNF31	Competitive combination	KRAS4B oncogenic mutation-driven non-small cell lung cancer (NSCLC)	USP25 promotes the progression of KRAS-driven NSCLC	Yang et al. (2025)
	PTEN	—	NSCLC	USP25 inhibits the proliferation of NSCLC	Zhu et al. (2021a)
​	TNKS1	Direct combination	Glioma	USP25 promotes the progression of glioma	Tang et al. (2022)
	STAT3	Direct combination	Ulcerative colitis (UC)	USP25 alleviates the progression of UC	Liu et al. (2025)
	NLRP3	Direct combination	Myocardial ischemia-reperfusion injury	USP25 improves MI/RI	Ye et al. (2025a)
	CRYAA	Direct combination	Diabetic retinopathy	USP25 exerts a photoreceptor protective effect	Sun et al. (2025)
	TXNIP	Direct combination	Osteoarthritis	USP25 exacerbates the progression of osteoarthritis	Sui et al. (2023)
Signal pathway	ERK	Activate	Tuberculosis	USP25 stabilizes B-Raf/C-Raf to activate the ERK pathway	Fu et al. (2025)
	JNK/p38 MAPK	Inhibition	Endotoxin Tolerance	USP25 inhibits the activation of the JNK/p38 MAPK inflammatory signaling pathway through TRAF3	Wen et al. (2019)
	NF-κB	Inhibition	AP	USP25 inhibits the activation of the NF-κB pathway through TBK1	Liu et al. (2022)
	Wnt/β-catenin	Activate	HCC	USP25 regulates the Wnt/β -catenin signaling pathway through TRIM21	Liu et al. (2023b)
	NF-κB/MAPK	Activate	DKD	USP25 inhibits the activation of NF-κB and MAPK pathways through TRAF6	Liu et al. (2023a)
	JNK/p38 MAPK	Inhibition	Obesity cardiomyopathy	USP25 blocks the activation of the JNK/p38 MAPK pathway by degrading TAK1	Ye et al. (2024)
	PI3K/AKT	Inhibition	PCOS	USP25 inhibits the activation of the PI3K/AKT pathway by stabilizing PTEN	Gao et al. (2021)
	Wnt/β-catenin	Activate	CRC	USP25 activates Wnt signaling by stabilizing TNKS1/2 and promoting Axin degradation	Xu et al. (2017)
	P53	Inhibition	Diffuse Large B-Cell Lymphoma	USP25 inhibits the activation of the p53 pathway through MDM2	Yang et al. (2023a)
	Wnt/β-catenin	Activate	Glioma	USP25 activates the Wnt/β-catenin pathway through TNKS1	Tang et al. (2022)
	TGFβ-SMAD	Inhibition	Hypertensive renal disease	USP25 blocks the activation of the TGFβ-SMAD pathway by inhibiting SMAD4	Zhao et al. (2023)
	IF-17-NF-κB/Jnk	Inhibition	Autoimmune diseases	USP25 inhibits IL-17 signaling via TRAF5/6	Zhong et al. (2012)

KIFC1, Kinesin family member C1; SERCA2a, Sarcoplasmic/endoplasmic reticulum (SR/ER) Ca (2+) ATPase, 2a; B-Raf, B-Raf proto-oncogene, serine/threonine kinase; TUG, Tether containing UBX, domain for GLUT4; TRAF3/6, TNF, receptor-associated factor 3/6; HBO1, Histone acetyltransferase binding to the origin recognition complex 1; APP, amyloid precursor protein; BACE1, Beta-site amyloid precursor protein cleaving enzyme 1; TBK-1, Tank-Binding-Kinase 1; KLF4, Kruppel-like factor 4; TRAF3, TNF, receptor-associated factor 3; BARD1, BRCA1-associated RING, domain 1; TAB2, TAK1-binding protein 2; RORγt, Retinoid-related orphan receptor gamma t; KEAP1, Kelch-like ECH-associated protein 1; NRF2, Nuclear factor erythroid-2-related factor 2; TRIM21, Tripartite motif-containing protein 21; Erlin1/2, ER, lipid raft-associated 1 and 2; PKM2, Pyruvate kinase M2; PTEN, phosphatase and tensin homolog; TNKS1/2, Tankyrase 1 and 2; MDM2, Murine double minute 2; RNF31, RING, finger protein 31; CRYAA, AlphaA-crystallin; TXNIP, thioredoxin interacting protein.

## 5 Specific role of USP25 in cardiovascular and cerebrovascular diseases

### 5.1 USP25 and cardiovascular diseases

#### 5.1.1 USP25 and cardiac hypertrophy

**FIGURE 5:**
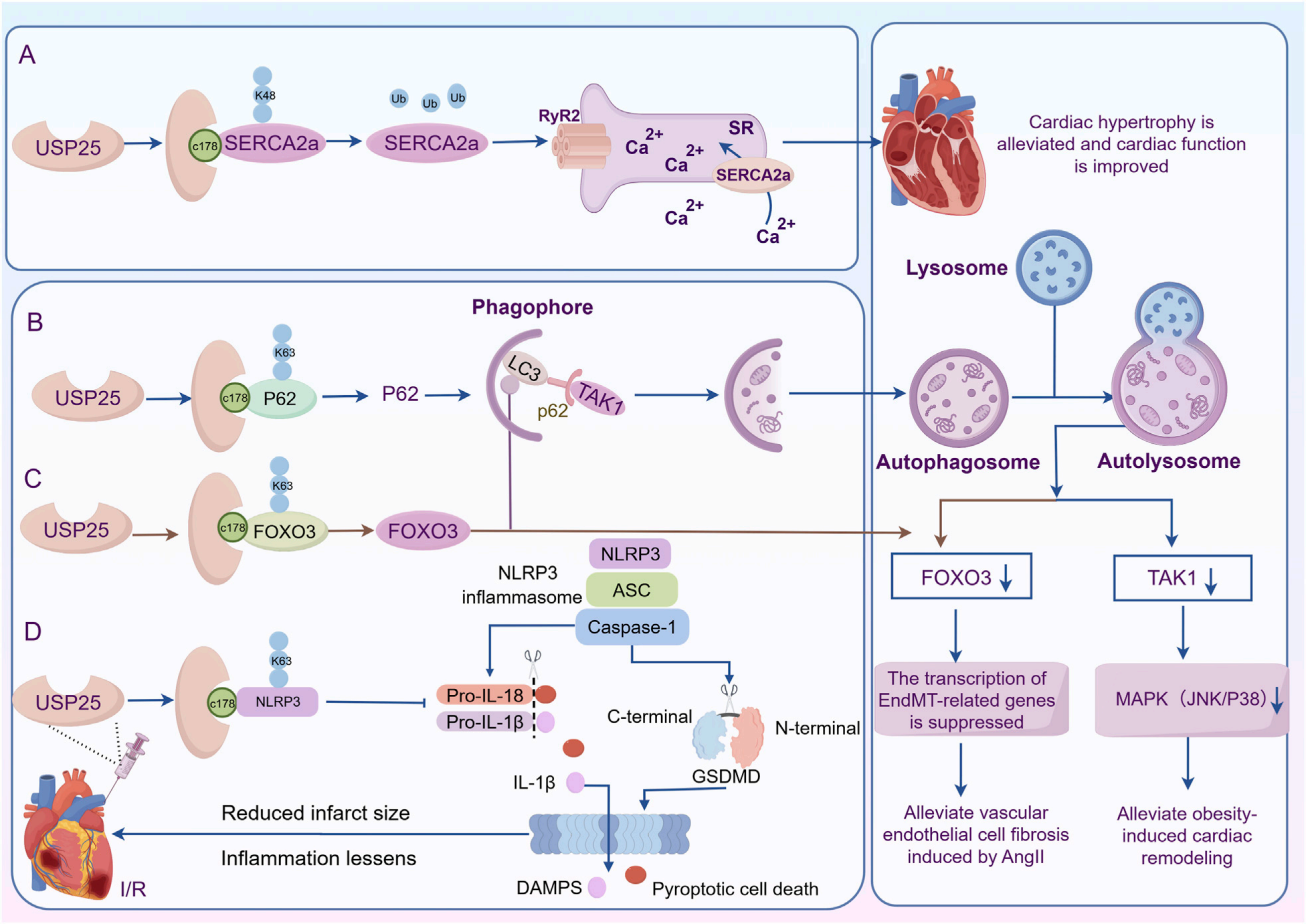
Mechanisms of USP25 in cardiac hypertrophy and ischemia–reperfusion (I/R) injury. **(A)** USP25 (C178 site) stabilizes the SERCA2a protein by removing the K48 ubiquitin chain of SERCA2a, maintains calcium homeostasis, and ultimately inhibits Ang II-induced cardiac hypertrophy. **(B)** USP25 alleviates obesism-induced cardiac hypertrophy by removing the K63 ubiquitin chain of P62, promoting the binding of P62 to TAK1 and its transport to autophagosomes, where TAK1 is degraded, leading to the inhibition of MAPK activation. **(C)** USP25 promotes the binding of FOXO3 to the autophagy marker protein LC3B by removing the K63 ubiquitin chain of FOXO3, degrading FOXO3 to inhibit EndMT-related genes and improve vascular remodeling. **(D)** USP25 effectively blocks the binding and oligomerization of NLRP3-ASC by clearing the K63 ubiquitin chain of NLRP3, thereby inhibiting the activation of NLRP3 inflammasome and pyroptosis of myocardial cells. SERCA2a, Sarcoplasmic/endoplasmic reticulum (SR/ER) Ca (2+) ATPase 2a; RYR2, Ryanodine receptor type 2; TAK1, Transforming growth factor beta-activated kinase 1; FOXO3, Forkhead box O3; EndMT, Endothelial-mesenchymal transition; NLRP3, NOD-like receptor protein 3; ASC, Apoptosis-associated speck-like protein; DAMPs, damage-associated molecular patterns.

Pathological cardiac hypertrophy constitutes a major precursor to heart failure and remains one of the leading global causes of mortality (Shimizu and Minamino, 2016; Nakamura and Sadoshima, 2018). Studies have shown that USP25 deficiency aggravates Ang II-, TAC-, and high-fat diet-induced cardiac hypertrophy, ventricular remodeling, fibrosis, and cardiac dysfunction, whereas AAV9-mediated restoration or overexpression of USP25 markedly ameliorates these pathological changes. The underlying mechanisms mainly include the following: first, USP25 stabilizes sarcoplasmic/endoplasmic reticulum (SR/ER) Ca (2+) ATPase 2a (SERCA2a), thereby maintaining cardiomyocyte calcium homeostasis and contractile function (Ye et al., 2023); second, USP25 binds to forkhead box O3 (FOXO3) and, in a Cys178-dependent manner, removes the K63-linked ubiquitin chain at lysine 258 of FOXO3, thereby enhancing FOXO3 interaction with LC3B and promoting its autophagic-lysosomal degradation, ultimately alleviating Ang II-induced vascular remodeling and fibrosis (Chen et al., 2025); third, USP25 directly interacts with P62 and transforming growth factor beta-activated kinase 1 (TAK1), and, through its catalytic cysteine residue at position 178, removes K63-linked ubiquitin chains from P62, thereby promoting TAK1 degradation via the autophagy-lysosome pathway, suppressing TAK1/MAPK-mediated inflammatory signaling, and attenuating cardiac remodeling (Ye et al., 2024). The related mechanisms are summarized in Figure 5. Building on these findings, Zhang et al. investigated whether the isoform USP25m plays a regulatory role in the heart. They proposed that in USP25^−/−^ mouse hearts, the absence of USP25m may aggravate cardiac pathology in part through its modulation of the glucose transporter GLUT4 (Zhang, 2024). Collectively, current evidence indicates that USP25 exerts cardioprotective effects against cardiac hypertrophy through a “triple axis of action”, calcium stabilization, anti-inflammation, and anti-apoptosis, mediated by stabilization of SERCA2a, degradation of TAK1, and inhibition of mitochondrial apoptosis. Notably, Ang II is not only a key stimulus driving cardiac hypertrophy and ventricular remodeling, but also one of the most commonly used pathogenic inducers in experimental models of hypertension. Therefore, the protective role of USP25 in Ang II-related models suggests that it may act as a shared regulatory node in hypertension-mediated cardiac remodeling and target-organ damage.

#### 5.1.2 USP25 and hypertension

Hypertension, one of the principal contributors to the global burden of cardiovascular disease, not only drives cardiac hypertrophy and ventricular remodeling through chronic pressure overload, but is also frequently associated with complications including renal fibrosis, vascular remodeling, and end-organ damage (Schmieder, 2010; Cai et al., 2021; Wu et al., 2023). Therefore, following the discussion of the relationship between USP25 and cardiac hypertrophy, further elaboration on the role of USP25 in hypertension-related vascular and renal injury would help provide a more comprehensive understanding of its protective significance in hypertension-associated cardiovascular remodeling.

Recent evidence has identified a protective role of USP25 in these pathological contexts. In Ang II-induced hypertensive mouse models, a significant upregulation of USP25 expression was observed in both renal and aortic tissues, suggesting its involvement in mitigating hypertension-mediated end-organ injury (Zhao et al., 2023; Chen et al., 2025). Mechanistic investigations further revealed that USP25 confers protection through two major pathways. First, it modulates the TGF-β–SMAD signaling cascade, thereby suppressing renal interstitial fibrosis and glomerulosclerosis (Zhao et al., 2023). Second, it activates the FOXO3–autophagy axis, facilitating the repair of vascular endothelial injury and preserving vascular homeostasis (Chen et al., 2025). This dual protective mechanism suggests that USP25 not only participates in the regulation of intracellular calcium homeostasis, inflammation, and apoptosis in cardiomyocytes, but may also indirectly alleviate cardiac afterload and myocardial remodeling by improving hypertension-related renal injury, vascular remodeling, and endothelial dysfunction. Therefore, USP25 may represent an important molecular node linking hypertension, vascular–renal injury, and cardiac hypertrophy. These findings also highlight USP25 as a potential therapeutic target for hypertensive nephropathy, vascular remodeling, and hypertension-associated cardiac injury.

#### 5.1.3 USP25 and myocardial ischemia-reperfusion injury (MI/RI)

Myocardial reperfusion remains the cornerstone of clinical management for acute myocardial infarction (Rentrop and Feit, 2015). However, reperfusion frequently induces MI/RI, resulting in myocardial cell death and worsening long-term prognosis (Zhang et al., 2024a). Using a MI/RI model in USP25-knockout mice, Ye et al. demonstrated that USP25 deficiency markedly aggravated ischemia–reperfusion–induced myocardial injury and accelerated pathological remodeling (Ye et al., 2025a). Mechanistically, USP25 directly interacts with NLRP3 and mediates K63-linked deubiquitination at the K243 residue through its catalytic site C178 (Ye et al., 2025a). This post-translational modification disrupts the interaction between NLRP3 and apoptosis-associated speck-like protein containing a CARD (ASC), prevents ASC oligomerization, and consequently inhibits NLRP3 inflammasome activation, ultimately attenuating myocardial pyroptosis (Ye et al., 2025a). These findings revealed a novel mechanism whereby USP25 confers myocardial protection against MI/RI through the “deubiquitination–inflammasome inhibition–pyroptosis suppression” axis. Notably, this study expanded on earlier work by Ye’s group exploring USP25 in obesity-related myocardial hypertrophy (Ye et al., 2024). Despite distinct initiating stimuli, ischemic stress and metabolic stress, both investigations demonstrated that USP25 protects the myocardium by regulating ubiquitination of different substrates (e.g., NLRP3 and P62). Collectively, this evidence supports the concept that USP25, as a “core molecule regulating ubiquitination”, exerts a protective role in diverse myocardial pathologies and provides a theoretical framework for the development of broad-spectrum USP25-targeted therapies for cardiomyopathy.

#### 5.1.4 USP25 and atherosclerosis (AS)

AS, the principal vascular lesion underlying myocardial infarction and ischemic stroke, is essentially a chronic progressive disease driven jointly by disordered lipid metabolism and macrophage-dominated chronic vascular inflammation (Shao et al., 2023a; Shao et al., 2023b; Shao et al., 2024). Recent studies have shown that USP25 expression is markedly reduced in atherosclerotic lesions and is predominantly localized in plaque macrophages (Su et al., 2026). In macrophages, USP25 directly cleaves K63-linked ubiquitin chains on the inflammatory switch protein RIPK1 through its catalytic residue C178, thereby blocking NF-κB inflammatory signaling, reducing macrophage inflammatory responses and foam cell formation, and ultimately suppressing atherosclerotic plaque growth. Current evidence supports USP25 as an important negative regulator of atherosclerosis and a potential new target for anti-inflammatory intervention. However, existing studies remain largely limited to animal models, and its cell-specific roles and clinical translational value still require further clarification.

### 5.2 USP25 in cerebrovascular injury and related brain diseases

In recent years, beyond the cardiovascular system, the role of USP25 in brain diseases has also attracted increasing attention (Li et al., 2023c; Munavar and Lenka, 2026; Xu et al., 2026). Current studies have mainly focused on cerebrovascular injuries such as ischemic stroke, as well as brain disorders associated with neuroinflammation and neurodegeneration.

#### 5.2.1 USP25 and ischemic stroke

Ischemic stroke accounts for more than 80% of all stroke cases and represents a leading cause of mortality and disability worldwide, with neuroinflammation recognized as a central mediator of secondary brain injury (Munavar and Lenka, 2026). In the mouse middle cerebral artery occlusion model, USP25 was specifically upregulated in microglia within the ischemic penumbra, whereas no significant changes were detected in neurons, suggesting that USP25 may exert a compensatory protective function through microglial activity (Li et al., 2023c). Mechanistically, USP25 attenuates excessive activation of the NF-κB and MAPK signaling pathways by removing K63-linked polyubiquitin chains from TAK1-binding protein 2 (TAB2), thereby reducing microglia-mediated proinflammatory responses and peripheral immune cell infiltration, ultimately alleviating ischemic brain injury. Further studies have shown that AAV9-mediated TAB2 knockdown not only mitigates cerebral ischemic injury but also abolishes the detrimental effects caused by USP25 deficiency, indicating that TAB2 is an important downstream target through which USP25 exerts its neuroprotective effects (Li et al., 2023c). It should be noted that, in ischemic stroke, microglia are not simply “harmful” or “beneficial,” but rather undergo dynamic and stage-dependent functional changes. In this context, USP25 primarily acts as an endogenous negative regulator that restrains excessive inflammatory responses, and its upregulation may represent a compensatory protective response to post-ischemic inflammatory imbalance. Therefore, the principal role of USP25 in stroke is not merely to suppress microglial activation, but to limit excessive proinflammatory microglial activation, thereby maintaining immune homeostasis in the brain and reducing secondary injury.

#### 5.2.2 USP25 and down syndrome-associated Alzheimer’s disease (DS-AD)

Beyond ischemic stroke, USP25 has also recently been implicated in other brain disorders closely associated with neuroinflammation and neurodegeneration, among which DS-AD is the most representative. DS results from trisomy of chromosome 21, on which the USP25 gene is precisely localized (Bull, 2020). This chromosomal localization suggests a potential involvement of USP25 in the pathological progression of DS. Cai et al. validated this hypothesis by generating USP25 transgenic overexpression mice (USP25-Tg). These mice exhibited abnormal cortical development, neurogenesis defects, and cognitive impairment, with underlying mechanisms linked to USP25 interference with the “cell cycle regulation–cyclin balance–neurogenesis inhibition–glial differentiation promotion” axis (Cai et al., 2023b). Notably, DS is the strongest risk factor for early-onset AD, and USP25 has been identified as a pivotal molecule connecting DS and AD. Zheng et al. established a DS–AD model by crossing 5×FAD AD mice with Dp16 DS mice and demonstrated that USP25 promotes proinflammatory cytokine release from microglia and enhances synaptic phagocytosis by stabilizing WD repeat and FYVE domain-containing protein 1 (WDFY1) and V0 sector of vacuolar proton ATPase (ATP6V0C), respectively (Zheng et al., 2021). This cascade induces neuroinflammation, synaptic damage, and cognitive impairment, thereby accelerating the pathological progression of DS-related AD (Zheng et al., 2021). Building on this work, Zheng’s group further investigated the regulatory role of USP25 in Aβ production, the central pathological driver of AD (Zheng et al., 2022). Their study revealed that USP25 stabilizes amyloid precursor protein (APP) and beta-site amyloid precursor protein cleaving enzyme 1 (BACE1), facilitates BACE1 localization to the Golgi apparatus, and promotes APP β-cleavage, thereby aggravating Aβ deposition and AD-related neuropathology (Zheng et al., 2022). Collectively, these findings indicate that USP25 initiates Aβ pathology through regulation of the APP/BACE1 pathway and amplifies neuroinflammation and synaptic damage via microglial activation, forming a synergistic pathogenic network from “source initiation” to “pathological amplification”. Additionally, the upstream regulation of USP25 has begun to be clarified. Li et al. demonstrated that the transcription factor SP1 binds to the USP25 promoter region and promotes its transcription. Inhibition of SP1 reduced USP25 expression, decreased Aβ production, and markedly improved DS- and AD-associated neuropathological phenotypes (Li et al., 2023b). In summary, as a key ubiquitination regulatory factor on chromosome 21, USP25 contributes to DS-related cognitive impairment and AD pathology through multidimensional mechanisms, neurogenesis regulation, Aβ metabolism, and neuroinflammation, and its expression is transcriptionally governed by specificity protein 1 (SP1).

Taken together, current evidence indicates that USP25 is deeply involved in brain-resident innate immune responses in both ischemic stroke and DS-AD, with its central role revolving around the regulation of neuroinflammatory intensity and microglial state transitions. This suggests that USP25 represents a functionally critical nodal molecule whose manipulation can produce clear phenotypic consequences. However, in contrast to its predominant role in limiting acute excessive inflammation in ischemic stroke, USP25 appears to promote pathological microglial responses and exacerbate neural injury in chronic neurodegenerative conditions such as DS-AD. Several factors may account for this discrepancy. First, the key substrates regulated by USP25 may differ across disease contexts. In stroke, TAB2 may serve as the dominant substrate, leading to an overall anti-inflammatory effect. In DS-AD, by contrast, substrates such as WDFY1, ATP6V0C, APP, and BACE1 may be more functionally relevant, thereby shifting the outcome toward enhanced inflammation, phagocytosis, and Aβ generation. Second, acute and chronic inflammatory conditions impose distinct demands on microglial programs. In stroke, USP25 may primarily regulate the “magnitude” of the inflammatory response, whereas in DS-AD it may instead shape the “state” of microglia. Third, different upstream stimuli may embed USP25 into distinct signaling networks. During stroke, microglia mainly respond to damage-associated molecular patterns and acute inflammatory signals released after ischemic necrosis. In DS-AD, however, microglia are chronically exposed to Aβ deposition, gene dosage imbalance, and a persistent inflammatory microenvironment, which more readily drives sustained activation, enhanced phagocytosis, and transcriptional reprogramming. These differences indicate that the function of USP25 is not intrinsically fixed as either protective or pathogenic; rather, it is more likely determined by the microglial activation program, the nature of upstream stimuli, and the spectrum of available substrates under a given disease condition. Therefore, therapeutic strategies targeting USP25 should take into full account the disease type, disease stage, and cerebral microenvironment, rather than simply treating USP25 as a unidirectional target for activation or inhibition.

## 6 Evaluation of the therapeutic potential of USP25 in cardiovascular and cerebrovascular diseases

### 6.1 Strategies for enhancing USP25 expression

The deficiency of USP25 expression constitutes a major contributing factor in the progression of cardiac diseases such as myocardial hypertrophy, hypertension, and MI/R. On this basis, two principal intervention strategies have been proposed: enhancing USP25 expression in the heart through exogenous delivery systems, or augmenting its endogenous expression via regulation of upstream targets. For exogenous supplementation, AAV9 serves as an optimal vector system for targeted cardiac expression of USP25, owing to its high transduction efficiency in cardiomyocytes and specificity of delivery. Although its technical feasibility, precise control of USP25 expression levels remains challenging. Excessive expression may disturb ubiquitination homeostasis and induce cytotoxicity, whereas insufficient expression fails to confer therapeutic benefit. Therefore, systematic dose-gradient studies are required to determine the optimal therapeutic window for USP25. Regarding endogenous promotion, multiple factors capable of regulating USP25 expression have been identified, including transcription factors, proteins, non-coding RNAs, and drugs (Table 2). Additionally, high-throughput drug screening platforms represent a promising strategy for identifying small molecules that selectively upregulate USP25 transcriptional activity or enhance its protein stability, whether from large-scale compound libraries or natural products. Notably, the regulation of USP25 activity depends not only on its expression level, but is also closely associated with its higher-order oligomerization state. Previous studies have suggested that USP25 can form a specific tetrameric assembly, a conformation associated with its autoinhibited or low-activity state (Zhou et al., 2023). Thus, the USP25 tetramer is not merely a structural form, but may represent a functionally constrained inactive state. From this perspective, targeting the interface of the inactive tetramer, disrupting the autoinhibitory assembly, and stabilizing the open or activated conformation may constitute a novel allosteric regulatory strategy distinct from traditional active-site ligands. It should be emphasized that this process essentially enhances the enzymatic activity of USP25 rather than upregulating its expression level. In the context of cardiovascular disease, if USP25 exerts protective deubiquitinating effects, then relieving its tetramer-mediated autoinhibition may help restore or enhance its function, thereby providing a theoretical basis for the development of novel cardiovascular therapeutics.

### 6.2 Strategies for inhibiting USP25 expression

In the pathological progression of diseases such as AD, tumors, and bacterial infections, abnormal activation or elevated expression of USP25 constitutes a major mechanism driving disease advancement. Inhibition of USP25 function can markedly mitigate pathological injury. Therefore, the development of specific USP25 inhibitors represents a promising therapeutic strategy. However, currently available USP25 inhibitors (e.g., AZ1, Vismodegib, FT20) generally display insufficient selectivity between USP25 and USP28, which substantially limits their translational potential. This feature may have dual implications depending on the disease context. If USP25 and USP28 are both upregulated and jointly promote disease progression, their simultaneous inhibition may enhance therapeutic efficacy and support the development of dual-target inhibitors. In contrast, if they exert distinct or even opposing functions, nonselective inhibition may reduce efficacy or increase adverse effects. Therefore, improving the selectivity of USP25 inhibitors remains critical for their clinical translation.

As discussed in Section 2, although USP25 and USP28 are highly homologous at the sequence and catalytic-domain levels, they differ in several aspects, including the AIM motif, autoinhibitory oligomerization, substrate recognition preference, and disease-associated functions. These differences provide important entry points for the development of selective USP25 inhibitors. Future drug design may focus on the USP25-specific AIM motif, the mechanism underlying its oligomeric state transition, substrate-interaction interfaces, and allosteric regulatory regions that differ from those of USP28. Compared with directly targeting the highly conserved catalytic center, allosteric inhibition directed at USP25-specific regulatory domains or protein–protein interaction interfaces may offer a more effective strategy for improving selectivity over USP28. In addition, determining high-resolution complex structures of USP25/USP28 bound to inhibitors is of considerable importance. Such structural information would not only help clarify the binding modes of existing inhibitors, the key interacting residues, and the molecular basis underlying insufficient selectivity, but also provide a foundation for subsequent structure-based drug optimization. Meanwhile, future studies should integrate proteomics, ubiquitinomics, CRISPR screening, and mass spectrometry to systematically define the substrate spectra of USP25 and USP28 across different diseases, cell types, and pathological stages, thereby further elucidating their disease-specific functions. Only by fully characterizing the respective substrates, signaling pathways, and functional distinctions of USP25 and USP28 can rational decisions be made between single-target and dual-target inhibition strategies. In summary, the development of USP25-targeted therapeutics faces challenges in selectivity, but also presents potential opportunities for dual-target intervention.

## 7 Conclusion and outlook

As an important member of the deubiquitinase family, USP25 has emerged as a central node in the shared pathological mechanisms of cardiovascular and cerebrovascular diseases by regulating protein stability, inflammatory signaling, and cell death pathways. Its roles in myocardial protection (e.g., SERCA2a stabilization, TAK1 degradation) and neural regulation (e.g., TAB2 deubiquitination, APP processing inhibition) highlight highly promising therapeutic targets for intervention in major cardiovascular and cerebrovascular disorders, including myocardial hypertrophy, heart failure, ischemic stroke, and AD. Therefore, USP25 is considered a highly promising novel target for the intervention of cardiovascular and cerebrovascular diseases. However, the therapeutic development of USP25 does not follow a simple logic of either “inhibition” or “activation”; rather, it exhibits a pronounced double-edged nature, which constitutes one of the central challenges in its clinical translation. On the one hand, in specific tissue contexts, USP25 helps suppress excessive inflammatory activation, maintain vascular barrier integrity, and buffer metabolic disturbances. Accordingly, loss of USP25 function or reduced activity may aggravate tissue injury and promote the progression of chronic diseases. On the other hand, in multiple types of cancer, USP25 may exert tumor-promoting effects by stabilizing oncogenic proteins, enhancing pro-survival signaling, or supporting remodeling of the tumor microenvironment. This implies that, without distinguishing disease type, tissue origin, molecular subtype, and disease stage, simply regarding USP25 as a universal inhibitory target may suppress tumors at the cost of disrupting its homeostatic functions in normal tissues; conversely, attempts to enhance the protective effects of USP25 may carry a potential risk of promoting tumorigenesis. Therefore, therapeutic intervention targeting USP25 must be based on precise stratification that is disease-specific, tissue-specific, and even temporally specific. In addition, the structural auto-inhibition of USP25 substantially increases the difficulty of drug development. Because its catalytic site is highly conserved across the USP family, traditional strategies targeting the catalytic center are prone to off-target effects, while its dynamic conformational transitions further constrain the design of highly selective molecules. Thus, the therapeutic development of USP25 depends not only on whether to intervene, but more importantly on how to achieve precise and selective modulation based on its conformational regulatory mechanisms. Furthermore, USP25 shares a high degree of structural similarity with its homolog USP28, which also represents a major bottleneck in the translation of targeted therapies. Notably, the rapid evolution of artificial intelligence technologies has provided new possibilities for overcoming these challenges.

In the future, integrating structural biology, computational chemistry, and precision delivery technologies may help improve the selectivity and druggability of USP25-targeting molecules, thereby promoting the clinical translation of innovative therapies based on precise deubiquitination regulation. Specifically, future research could focus on three main directions: the construction of high-resolution expression atlases, mechanistic investigation, and the development of targeted therapeutics. On the one hand, single-cell RNA sequencing can be used to characterize the expression patterns of USP25 across different cell types in the heart, vasculature, and brain, as well as to define disease-associated transcriptional landscapes. In combination with spatial transcriptomics, this approach would further reveal the spatial distribution of USP25 under physiological and pathological conditions, together with microenvironment-dependent alterations. On the other hand, further studies are needed to elucidate the regulatory mechanisms governing USP25 nucleocytoplasmic shuttling, map its spatiotemporally defined substrate network, and clarify the dynamic functional changes of USP25 across different stages of cardio-cerebrovascular diseases and among distinct cell types. Additionally, the development of highly selective USP25 activators and inhibitors represents a key breakthrough in this field. Strategies for developing USP25 activators mainly involve exogenous and endogenous supplementation, whereas the core approaches for developing USP25 inhibitors can be explored from the following four aspects. (i) Leveraging the unique AIM motif of USP25 (absent in USP28) to design peptide inhibitors that stabilize USP25 in a tetrameric autoinhibitory state, thereby achieving highly selective inhibition. (ii) Building on inhibitors targeting full-length USP25/USP28, simultaneously developing antagonists against USP25 truncation variants to minimize drug toxicity in normal tissues. (iii) Designing inhibitors that block interactions between USP25 and its binding partners, thereby impairing its pathological functions by disrupting intermolecular interaction networks. (iv) Investigating the dynamic binding processes between USP25/USP28 and inhibitors, elucidating inhibitor-induced conformational changes, and providing a molecular framework for rational optimization of inhibitor structures. These research efforts are essential not only for elucidating the precise mechanisms of USP25 in cardiovascular and cerebrovascular diseases but also for establishing a robust theoretical basis for innovative therapies that precisely modulate USP25 activity in defined subcellular pools, including selective agonists/inhibitors and strategies regulating its nucleocytoplasmic shuttling.
